# Human SETMAR is a DNA sequence-specific histone-methylase with a broad effect on the transcriptome

**DOI:** 10.1093/nar/gky937

**Published:** 2018-10-17

**Authors:** Michael Tellier, Ronald Chalmers

**Affiliations:** School of Life Sciences, University of Nottingham, Queen's Medical Centre, Nottingham NG7 2UH, UK

## Abstract

Transposons impart dynamism to the genomes they inhabit and their movements frequently rewire the control of nearby genes. Occasionally, their proteins are domesticated when they evolve a new function. SETMAR is a protein methylase with a sequence-specific DNA binding domain. It began to evolve about 50 million years ago when an Hsmar1 transposon integrated downstream of a SET-domain methylase gene. Here we show that the DNA-binding domain of the transposase targets the enzyme to transposon-end remnants and that this is capable of regulating gene expression, dependent on the methylase activity. When SETMAR was modestly overexpressed in human cells, almost 1500 genes changed expression by more than 2-fold (65% up- and 35% down-regulated). These genes were enriched for the KEGG Pathways in Cancer and include several transcription factors important for development and differentiation. Expression of a similar level of a methylase-deficient SETMAR changed the expression of many fewer genes, 77% of which were down-regulated with no significant enrichment of KEGG Pathways. Our data is consistent with a model in which SETMAR is part of an anthropoid primate-specific regulatory network centered on the subset of genes containing a transposon end.

## INTRODUCTION

Transposable elements (TEs) are almost ubiquitous and their transposases are the most abundant genes in nature (1). Because the genetic information encoded by TEs is usually used only for their own survival, they have been considered as selfish genomic-parasites (2). However, it is now clear that TEs are also an important source of genetic novelty (3). For example, they promote the emergence of new gene regulatory networks by dispersing transcription factor binding site in the genome and they give rise to new microRNAs and long intergenic non-coding RNAs (4–9). A less frequent event is exaptation, when a TE contributes sequences to a new *bona fide* host protein and evolves a new function (6). One of the best examples is V(D)J recombination in the vertebrate immune system (10). In this case, the RAG1 recombinase and the recombination signal sequences preserve almost all of the respective functions of the ancestral transposase and its cognate binding sites in the transposon ends (inverted terminal repeats, ITRs). The human CSB-PGBD3 protein, which arose from the domestication of a piggyBac transposon, has been shown to affect gene expression (11–13). Although it can still bind to remnants of the ancestral transposon ends, its regulatory activity is at least partly mediated by an interaction with the AP-1 transcription factor. The precise functions of the other ∼50 domesticated transposase proteins in the human genome remain unknown (14).

**Figure 1.**
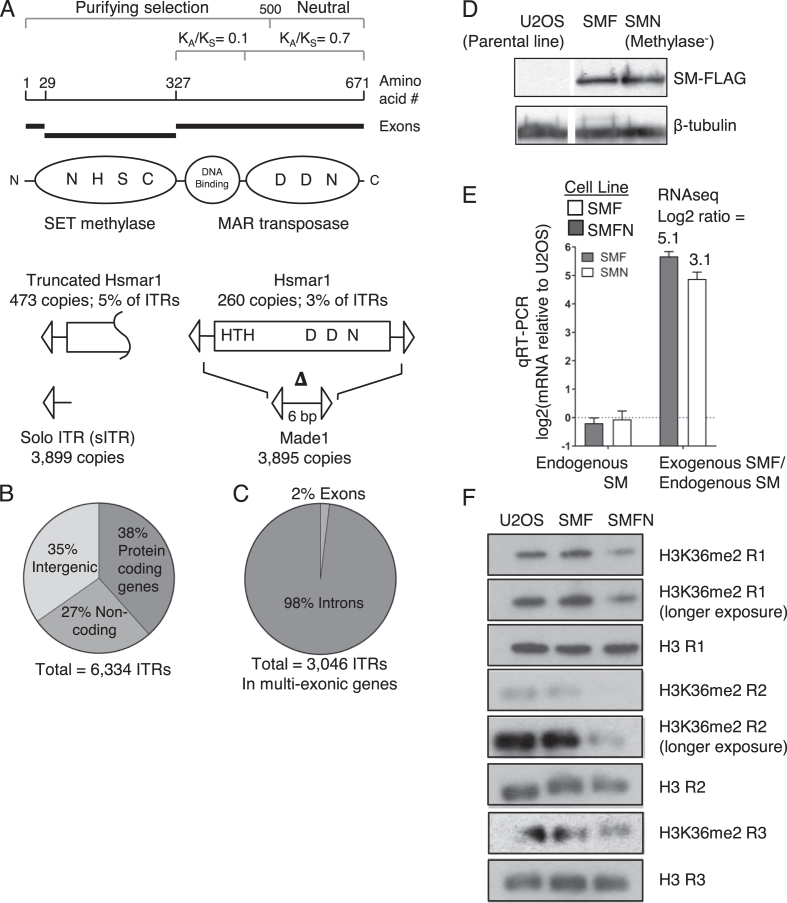
Hsmar1 remnants in the human genome and expression of ectopic SETMAR. (**A**) The SETMAR domain and exon structure are illustrated together with the helix-turn-helix (HTH) DNA binding motifs and key active site residues in the methylase and transposase active sites. The third D residue (aspartate) that coordinates the catalytic metal ion is an N (asparagine) in SETMAR. All 260 full-length copies of Hsmar1 have inactivating point mutations and indels. Made1 elements comprise of six bp flanked by a pair of ITRs. (**B** and **C**) Distribution of the 6,334 ITRs in the human genome. (**D**) A Western blot for the FLAG-tagged codon optimized SETMAR in the U2OS, SMF and SMFN cell lines. SMFN has the N210A substitution of an essential residue in the SETMAR methylase domain active site. (**E**) qRT-PCR of the endogenous and the transgenic SETMAR in the SMF and SMFN cell lines. Figures above rightmost columns are the ratios of expression derived from the RNA-seq experiments presented in Figure 3. (**F**) Western blot of the indicated cell lines using an H3K36me2 antibody and a histone H3 antibody as a loading control. The lanes probed for H3 and H3K36me2, respectively, were from a single gel loaded with the same amount of protein. The same result was obtained from three biological replicates (R1 to R3) performed on different batches of cells.

The human SETMAR protein is expressed in most tissues and cells and is a fusion between a SET-domain protein methylase and the Hsmar1 transposase (Figure 1A) (15). Exaptation occurred in the anthropoid primate lineage between 40 and 58 million years ago, during a period when many key genetic changes and adaptations were taking place (16). In the region of the SETMAR gene encoding the transposase DNA-binding domain the ratio of nonsynonymous (*K*_A_) to synonymous (*K*_S_) nucleotide substitutions is 0.1 (Figure 1A). This indicates that the domain is under purifying selection and therefore has a function. The *K*_A_/*K*_S_ ratio for the region encoding the transposase catalytic domain is 0.7, which indicates that it is drifting (16). The Hsmar1 transposon itself is currently inactive in humans due to genetic drift but functional orthologs of the SET gene are deeply conserved in the mammalian and avian lineages.

Reports have linked SETMAR to several cellular processes including non-homologous end joining (NHEJ) (17), integration of lentiviruses and transfected plasmids (17,18), restart of stalled replication forks and chromosomal decatenation (19). Its role in these processes was attributed to the SET domain, which was shown to methylate histone H3 lysine 36 (H3K36me), and the catalytic domain of the transposase, which was shown to cleave branched DNA (17). On a genome-wide scale, H3K36me is one of the most abundant epigenetic marks and has been linked with active and inactive chromatin, transcriptional elongation and repression, DNA replication and repair, alternative splicing, dosage compensation and oncogenic transformation (20–23). However, it should be noted that these reports include observations in distantly related organisms that lack SETMAR. Thus, it is difficult to interpret the meaning of an abundant histone mark, such as H3K36me, which depends on its location and its respective readers, writers and erasers.

Another reason that the cellular roles of SETMAR remains obscure is that there is some uncertainty regarding its methylase and nuclease activities. Two groups presented *in vitro* evidence that the nuclease activity of the transposase has been largely abolished by a substitution of the third essential D residue in the active site and other mutations (24,25). A third study failed to reproduce nucleosome methylation *in vitro* but used proteomics to detect methylation of the splicing factor snRNP70 by SETMAR (26). The evolutionary conservation of the DNA binding domain of SETMAR rather than its catalytic domain also suggests that the Hsmar1 ITR binding activity played an important role in SETMAR evolution. If SETMAR was acting primarily as a structure-specific DNA repair endonuclease, the DNA binding domain might be expected to evolve neutrally.

In an attempt to clarify the cellular role of SETMAR, we have tested an evolutionary conjecture that was proposed to account for the fact that the majority of transposon exaptation events in higher eukaryotes involve DNA transposons, despite their paucity of numbers compared to the retro-elements. The hypothesis is that the DNA-binding domain of the transposase, which is absent in endogenous retro-elements, is easily reused by the host (6). This hypothesis predicts that the DNA binding domain of SETMAR serves to target the protein to a subset of the Hsmar1 ITRs dispersed throughout the human genome. This is attractive because it incorporates roles for the DNA binding domain and its cognate binding sites in the ITRs of the ancestral transposon. We have addressed the hypothesis using ChIP-exo and RNA-seq on stable cell lines over-expressing a modest amount of wild-type or a methyltransferase-deficient SETMAR. We find that SETMAR binds to ITRs located in Hsmar1 remnants *in vivo* and regulates the expression of many genes containing an ITR. Regulation is largely dependent on the methyltransferase activity. These observations suggest that the fusion of the ancestral transposase and SET genes in anthropoid primates might have perturbed the transcriptome significantly and could have contributed to the emergence of new regulatory relationships between genes.

## MATERIALS AND METHODS

### Plasmids

An artificial codon-optimized version of SETMAR was synthesized by Gene Art (Thermo Fischer) and cloned into pcDNA4TO at the EcoRI/NotI restriction sites. The significant differences between the nucleotide sequences of the endogenous and exogenous SETMAR genes allowed them to be distinguished in the RNA-seq experiments. The N210A mutation in the methylase active site was introduced by PCR.

### Stable transfection of T-Rex-U2OS cells

For each transfection, 2.5 × 10^5^ of cells were seeded in a 6-well plate and grown overnight in DMEM supplemented with 10% FBS. The plasmids were transfected using Lipofectamine 2000 (Invitrogen), following manufacturer's instruction. After 24 h, a quarter of the cells were transferred to 100 mm dishes and the medium supplemented with 400 μg/ml of zeocin (Invivogen). After 2 weeks of selection, single foci were picked and grown in a 24-well plate. The expression of the gene of interest was verified in each cell line by inducing the PCMV promoter with doxycycline at a final concentration of 1 μg/ml for 24 h.

### Western blotting

Whole cell extracts were harvested from cultures at ∼90% confluency in six-well plates. Briefly, cells were washed two times with ice-cold PBS then pelleted for 5 min at 3000 × g at 4°C. Samples were resuspended in 100 μl of Radio ImmunoPrecipitation Assay (RIPA) buffer (10 mM Tris–HCl pH8.0, 150 mM NaCl, 1 mM EDTA, 0.1% SDS, 1% Triton X-100, 0.1% sodium deoxycholate) with freshly added protease inhibitor cocktail (Roche Applied Science) and incubated on ice for 30 minutes, with a vortexing every 10 min. Cell lysates were centrifuged for 15 min at 14 000 × g at 4°C and the protein in the supernatants was quantified by the Bradford assay.

Histones were harvested from cultures at ∼90% confluency in 6 cm dishes. Briefly, cells were washed two times with ice-cold PBS and pelleted for 5 min at 3000 × g at 4°C. Cells were resuspended in Triton Extraction Buffer (TEB) (PBS, 0.5% Triton X-100 (v/v), 2 mM PMSF, 0.02% NaN3 [v/v]) at a density of 10^7^ cells/ml. Cells were lysed for 10 min on a rotor at 4°C, followed by centrifugation at 400 × g for 10 minutes at 4°C. The supernatant was removed and the cells washed with half the volume of TEB, followed by centrifugation at 400 × g for 10 min at 4°C. The supernatant was removed and the pellet resuspended in 0.2 M HCl at a cell density of 4 × 10^7^ cells per ml. The histones were acid extracted overnight at 4°C on a rotor. The extract was centrifuged at 400 × g for 10 minutes at 4°C and the supernatant recovered. Protein concentration was determined using the Bradford assay. Protein, 2 or 20 μg, was mixed with 2X SDS loading buffer, boiled for 5 minutes, and electrophoresed on a 10 or 15% SDS-PAGE gel. Proteins were transferred to a polyvinylidene difluoride (PVDF) membrane, which was blocked in 5% milk or BSA (Roche) and incubated with specific primary antibodies at 4°C overnight. After washing, membranes were incubated with horseradish peroxidase (HRP)-conjugated secondary antibodies for one hour at room temperature, washed, and signals were detected with the ECL system (Promega) and Fuji medical X-ray film (Fujifilm).

The following antibodies were used: anti-histone H3 (rabbit IgG, 1:10 000 dilution, ab1791, Abcam), anti-histone H3K36me2 (mouse IgG, 1:2000 dilution, 61019, Active Motif), anti-beta Tubulin (rabbit polyclonal IgG, 1:500 dilution, ab6046, Abcam), anti-FLAG (rabbit, 1:500 dilution, F7425, Sigma). The secondary antibodies were horseradish peroxidase-conjugated anti-mouse (goat polyclonal, 1:10 000 dilution, 12-349, Merck) and anti-rabbit (goat polyclonal, 1:5000-1:10 000, ab6721, Abcam).

### Transcriptomes acquisition and analysis

Total RNAs were isolated from cells grown to ∼90% confluency in six-well plate with the High Pure RNA isolation kit (Roche Applied Science), following manufacturer's instructions. The samples were quantified with a Nanodrop Spectrophotometer and their quality verified with a Bioanalyzer (Agilent). Only samples with a RIN number >9 were used. Illumina TrueSeq RNA Sample Preparation v2 was used according to manufacturer's instructions. Indexed samples were sequenced on an Illumina NextSeq 500 machine to generate 2 × 75 bp reads. The adapters and the low quality sequences were trimmed with Sythe and Sickle. Reads were first filtered against rRNA and tRNA sequenced and then mapped to the hg19 human genome assembly using Tophat2 (27) version 2.1.0 with default options. RNA-seq was performed on biological duplicates with a total number of mapped reads between 70 and 157 million paired end reads. Protein-coding genes were obtained from the ENSEMBL GRCh37.75 GTF file. For calculating the fragments per kilobase of transcript per million mapped reads (FPKM) values, exons were extracted in R with the GenomicFeatures package and grouped by ‘gene’. Expression at the transcript level was quantified with HTSeq version 0.6.1 and differential expression was calculated with the DESeq2 software version 3.2, keeping only the genes with a fold change <−2 or >2 and an adjusted *P*-value of 0.05. RNA-seq smear plots showing average gene expression (x-axis) versus log_2_ fold change in gene expression were produced with GraphPad Prism 7.02.

### qRT-PCR

U2OS cells were harvested from cultures at ∼90% confluency in six-well plates. Briefly, cells washed twice with ice-cold PBS and pelleted for 5 min at 3000 × g at 4°C. Cells were resuspended in 1 ml of TRIzol (ThermoFisher Scientific) and incubated at room temperature for 5 min. 200 μl of chloroform was added to each lysate, vortexed for 15 s and incubated at room temperature for 3 min. The lysates were centrifuged at 12 000 × g for 15 min at 4°C and the upper phase transferred to a new tube. 500 μl of isopropanol was added to each tube, mixed gently, incubated at room temperature for 10 min then centrifuged at 12 000 × g for 15 min at 4°C. After removal of the supernatant, 1 ml of 75% ethanol was added to the pellet, vortexed and centrifuged at 7500 x g for 5 min at 4°C.The pellets were air-dried briefly and resuspended with 20 μl of RNase-free water. After incubating the samples for 10 min at 55°C, 70 μl of RNase-free water, 10 μl of DNase buffer and 1 μl of DNase enzyme were added to each tube and incubated for 30 min at 37°C. A phenol-chloroform pH4.2 extraction was performed on each sample and the RNA pellet resuspended in 20 μl of RNase-free water. Total RNA (500 ng) was converted to cDNA with random hexamers and the SuperScript III kit (Invitrogen), according to the manufacturer's instructions. cDNA was amplified by qPCR with a QuantiTect SYBR Green PCR kit (QIAGEN) and a Rotor-Gene RG-3000 (Corbett Research). For each reaction, the following components were included: 1 μl of template, 1 μl of primer pair mix (10 μM), 3 μl of water and 5 μl of SYBR Green Mix (2×). The thermo-cycling parameters were: 95°C for 15 min followed by 40 cycles of 94°C for 15 s, 57°C for 20 s and 72°C for 25 s. The Roto-Gene Q Series Software was used to calculate the threshold cycle (Ct) value. Signals are presented as a percentage of Input DNA after removal of the IgG background signal. The primers were designed with Primer3Plus (https://primer3plus.com/cgi-bin/dev/primer3plus.cgi) and the NCBI Primer-BLAST tool (https://www.ncbi.nlm.nih.gov/tools/primer-blast/) to verify the specificity of the primers. Results were represented as fold change, normalized to TUBB. The sequences of primers used for qRT-PCR are given in Supplementary Table S4. Experiments were replicated at least three times to ensure reproducibility, and each RNA sample was measured in triplicate by qPCR.

### ChIP-qPCR and ChIP-exo

ChIP was performed as previously described (28). Briefly, U2OS cells were grown in 150 mm dishes until they reached 80–90% confluency (∼1 × 10^7^ cells). The cells were then fixed with 1% formaldehyde for 10 min at room temperature with shaking. Formaldehyde was quenched with 125 mM glycine and incubated for 5 min at room temperature with shaking. Cells were placed on ice and washed twice with ice-cold PBS. Cells were scraped in ice-cold PBS and transferred to a fresh chilled Eppendorf, then centrifuged for 10 min at 1500 rpm at 4°C. The cells were resuspended in ChIP lysis buffer (10 mM Tris–HCl ph8.0, 0.25% Triton X-100, 10 mM EDTA, and protease inhibitor cocktail) and incubated for 10 min on ice. Lysis buffer was removed by centrifugation for 5 min at 1500 × g at 4°C and nuclear pellets were resuspended in ChIP Wash buffer (10 mM Tris–HCl pH8.0, 200 mM NaCl, 1 mM EDTA, protease inhibitor cocktail). Wash buffer was removed by centrifugation for 5 min at 1500 × g at 4°C and nuclear pellets were resuspended in ChIP Sonication buffer (10 mM Tris–HCl pH 8.0, 100 mM NaCl, 1 mM EDTA, protease inhibitor cocktail). Cells were sonicated twice for 15 min at high amplitude, 30 s ON/30s OFF on a Bioruptor (Diagenode). This was followed by centrifugation at 13 000 rpm for 15 min at 4°C, and the supernatant was transferred to a fresh Eppendorf.

Ten microliter of Protein G Dynabeads per immunoprecipitation (IP) were washed with 100 μl of RIPA buffer. 25 μg (for histones) or 80 μg (for SETMAR) of chromatin was added and left to shake for 30 min at 4°C. The supernatant was recovered and the beads discarded. 1 μg of antibody (Flag M2 (F1804, Sigma), histone H3 (ab1791, Abcam), histone H3K4me3 (ab8580, Abcam), histone H3K36me2 (60019, Active Motif), histone H3K36me3 (ab9050, Abcam), RNA polymerase II (NBP2-32080, Novus Biologicals), normal rabbit IgG (sc-2027, Santa Cruz Biotechnology), normal mouse IgG (sc-2025, Santa Cruz Biotechnology) was added and mixed overnight on a rotor at 4°C. 15 μl of Dynabeads per IP were washed in 100 μl RIPA buffer. The beads were saturated with 15 μl RIPA containing 4mg/ml of bovine serum albumin (BSA) and mixed overnight on a rotor at 4°C.

The blocking solution was removed from the beads and mixed with the sonicated extract incubated with antibody. After 1-hour incubation on a rotating wheel at 4°C, IgG supernatant was retained as total input. Beads were washed three times with 300 μl ice-cold RIPA, three times with 300 μl ice-cold High Salt Wash Buffer (10 mM Tris–HCl pH8.0, 500 mM NaCl, 1 mM EDTA, 0.1% SDS, 1% Triton X-100, 0.1% sodium deoxycholate), two times with 300 μl ice-cold LiCl Wash buffer (10 mM Tris–HCl pH8.0, 250 mM LiCl, 1 mM EDTA, 1% NP-40, 1% sodium deoxycholate) and two times with 300 μl TE (10 mM Tris–HCl pH 7.5, 1 mM EDTA). For each IP sample, 50 μl Elution buffer (100 mM NaHCO3, 1% SDS, 10 mM DTT) was added and mixed for 15 min at 25°C at 1400 rpm with shaking. The elution was repeated once and both elutes combined. For each input sample, 90 μl Elution buffer (containing 10 mM DTT) was added to 10 μl total input.

RNase A (0.5 μl of 10 mg/ml) was added to each sample and incubated for 30 min at 37°C. 200 mM NaCl was added followed by incubation for 5 h at 65°C to reverse the crosslinks. 2.5× volume of 100% ethanol was added and incubated overnight at -20°C. The ethanol was removed after centrifugation for 20 minutes at 13000 rpm at 4°C. The pellets were resuspended in 100 μl TE and 25 μl of 5× Proteinase K buffer (50 mM Tris–HCl pH 7.5, 25 mM EDTA, 1.25% SDS) and 1.5 μl Proteinase K (20 mg/ml) were added to each sample. These were incubated for 2 h at 45°C to degrade the proteins. DNA was purified using Qiagen PCR Purification Kit and kept at −20°C.

ChIP samples were analyzed by real-time qPCR with a QuantiTect SYBR Green PCR kit (QIAGEN) and a Rotor-Gene RG-3000 (Corbett Research). For each reaction, the following components were included: 1 μl of template, 1 μl of primer pair mix (10 μM), 3 μl of water and 5 μl of SYBR Green Mix (2×). The thermo-cycling parameters were: 95°C for 15 min followed by 40 cycles of 94°C for 15 s, 57°C for 20 s and 72°C for 25 s. The Roto-Gene Q Series Software was used to calculate the threshold cycle (Ct) value. Signals are presented as a percentage of Input DNA after removal of the IgG background signal. The primers were designed with Primer3Plus (https://primer3plus.com/cgi-bin/dev/primer3plus.cgi) and the NCBI Primer-BLAST tool (https://www.ncbi.nlm.nih.gov/tools/primer-blast/) to verify the specificity of the primers. The sequences of primers used for ChIP-qPCR are given in Supplementary Table S4. Experiments were replicated three times and each ChIP sample was measured in triplicate by qPCR.

For ChIP-exo experiments, approximately 15 million cells of the SMF cell line were fixed with 1% formaldehyde for 10 min at room temperature and quenched with 0.125 M glycine for 5 min at room temperature. The cells were washed twice with ice-cold PBS and resuspended in 0.5 ml of Cell lysis buffer (10 mM Tris pH 8.0, 10 nM NaCl, 0.5% NP40) supplemented with fresh Complete Protease Inhibitor (CPI) cocktail (Roche Applied Science) and incubated for 10 min on ice. The nuclei were pelleted for 5 min at 660 × g at 4°C and washed once with 1 ml of ice-cold PBS. Nuclei pellets were resuspended in 1 ml of room temperature Nuclei Lysis Buffer (50 mM Tris pH 8.0, 10 mM EDTA, 0.32% SDS) supplemented with fresh CPI cocktail and incubated for 10 min on ice. The nuclear lysates were transferred to a fresh, ice-cold 15 ml Falcon tube and the Eppendorf tube washed with 0.6 ml of cold IP Dilution Buffer (20 mM Tris pH 8.0, 2 mM EDTA, 150 mM NaCl, 1% Triton X-100) complemented with fresh CPI cocktail and combined with the nuclear lysates. Chromatin was sheared to ∼ 300 bp fragments with a Bioruptor (Diagenode) using the following conditions: power setting: high, time setting: 15 cycles of 30 s ‘on’/30 s ‘off’ during two sessions of 15 min. The sonicated extracts were transferred to fresh, ice-cold Eppendorf tubes and centrifuged for 10 min at 20 800 × g at 4°C. The resulting sheared chromatin and the anti-FLAG M2 (Sigma) antibody were sent to the Peconic company (PA, USA) for further processing.

### Computational analyses

#### Hsmar1 remnants and ITRs in the human genome

Hsmar1 remnants locations were extracted from the output of the RepeatMasker (RM) Genomic Datasets produced for the human genome, hg19 assembly, RM 3.3.0 track, Repbase libraries 20120124. Hsmar1 transposon ends were obtained using the BLAST software against the human genome, hg19 assembly, and the outputs parsed for transposon ends retaining at least 80% of the length and identity of the ancestral Hsmar1 ITR. Intragenic ITRs were obtained by intersecting the list of ITRs and the set of protein-coding genes from the ENSEMBL GRCh37.75 GTF file.

#### ChIP-exo

The sequences were mapped against the human genome version GRCh37 (hg19) using BWA (29) version 0.7.5a with default parameters, and BAM-formatted files were created using Samtools (30) version 1.2. Mapped reads were then de-duplicated using Picard to remove PCR duplicates. The bam files from both biological replicates were then merged and MACS (31) version 1.3.7.1 with default parameters was used for peak calling. De novo motif discovery on the peaks was performed with the MEME suite MEME-ChIP (32) using default algorithm parameters.

#### Nucleosome and histone methylation data

The published datasets for H3K9ac and input reads in U2OS were obtained from Encode (33) under the accession number GSE31755. Nucleosome data for U2OS were obtained from (34) under the accession number GSE71577. Nucleosome data for H1 and H9 were obtained from (35) under the accession number GSE49140. Nucleosome data for GM18508 was obtained from (36) under the accession number GSE36979. Adapters were trimmed with Cutadapt v. 1.9.1 (37). All sequences were mapped using Bowtie2 v. 2.2.5 (38) against the human genome (GRCh37 hg19 from Ensembl) with up to two mismatches allowed. Bam-formatted files were created using SAMtools v. 1.3.1 (30) and only uniquely mapped reads were kept. The PCR-duplicates were removed using Picard. The total number of reads for each sample was then normalized to a 1× depth of coverage (nucleosomes) of the human genome or in RPKM (H3K9ac and Input) with deepTools2 v. 2.2.4 (39). Metagene profiles were generated using deepTools2 computeMatrix tool with a bin size of 10 bp and the plotting data obtained with plotProfile –outFileNameData tool. Graphs (Input signal subtracted from IP signal) were then created with GraphPad Prism 7.02.

#### *P*-values and significance tests

*P*-values for hypergeometric distribution were computed with Microsoft Excel. The hypergeometric distribution is used to determine whether a sub-population, in this case the genes with an ITR, are over- or under-represented in a sample, in this case the genes differentially expressed or with a ChIP peak. Unpaired *t*-test and Mann–Whitney U test were performed in GraphPad Prism 7.02.

## RESULTS

Studies linking SETMAR to DNA recombination and repair have invoked direct roles for the methylase domain and the catalytic domain of the transposase. However, the *K*_A_/*K*_S_ profile across the gene suggest that the DNA binding domain of the transposase is more important than the catalytic domain, which is under relaxed selection. We therefore set out to test the hypothesis that the primary function of SETMAR is mediated by the targeting of the protein to a subset of the 7000 Hsmar1 remnants dispersed throughout the human genome (Figure 1A). Almost half of the remnants are Made1 elements, which are miniature-transposons comprised of 6 bp flanked by a pair of ITRs (Figure 1A). About 500 of these are annotated as miRNAs or miRNA-like (5). Overall, within the 7000 Hsmar1 remnants we found 6334 ITRs that still have at least 80% of the length and identity to the canonical 28 bp ITR sequence (Supplementary Table S1 and Methods section). About two-thirds of these ITRs are located in non-coding genes or the introns of protein coding genes (Figure 1B, C), perhaps reflecting preferential integration into transcribed regions (40,41).

Initial transient transfection experiments in osteosarcoma cells (U2OS) with SETMAR downstream of a CMV promoter gave an overexpression of 2500-fold. We therefore established stable cell lines expressing modest levels of the protein downstream of a repressed CMV promoter. SETMAR was FLAG-tagged (SMF) and codon optimized so that the endogenous mRNA could be distinguished in RNA-seq experiments. A mutant version had a single inactivating point mutation (N210A) in the key NSHC motif of the methylase active site (SMFN) (17,42). Transgene overexpression was in the range of 8- to 32-fold higher than endogenous SETMAR as measured by western blotting, RNA-seq and qRT-PCR, and did not affect the expression of the endogenous SETMAR gene (Figure 1D, E). We also used Western blotting to test whether overexpression of SMF and SMFN affected the amount of H3K36me2 in a histone extract (Figure 1F and Supplementary Figure S1A). There was a significant loss in the SMFN cell extract, indicating that the methylase mutant may have a dominant-negative phenotype. However, there was only a slight increase in methylation in the SMF cell extract. The absence of a clear increase might be due to the high background of H3K36 dimethylation in human cells, which is estimated at 30–50% of the histone H3 (43,44).

### SETMAR binds Hsmar1 ITRs *in vivo*

**Figure 2.**
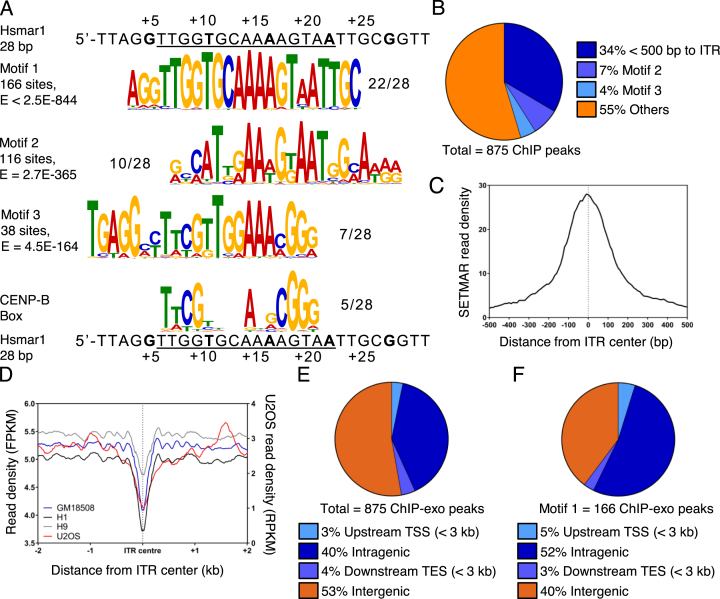
SETMAR binds Hsmar1 ITRs and other sequences *in vivo*. (**A**) SETMAR binding *in vivo* was assessed by ChIP-exo and enriched motifs were identified with the MEME-ChIP software. The three most enriched motifs are presented: Motif 1 corresponds to the SETMAR binding site on the Hsmar1 ITR; Motif 2 is a degenerate ITR sequence associated with a subset of Made1 elements; Motif 3 resembles an ITR and is enriched in centromeric regions of the genome. CENP-B box and the consensus Hsmar1 ITR are shown below. (**B**) Distribution of the ChIP-exo peaks with respect to annotated Hsmar1 ITRs. (**C**) Metaprofile of ChIP-exo reads around the center of the 419 bound ITRs. (**D**) MNase-seq nucleosome metaprofiles around annotate Hsmar1 ITRs were generated from the ENCODE data for U2OS, a lymphoblastoid cell line (GM18508), and two human embryonic stem cell lines (H1 and H9). (**E** and **F**) Distribution of SETMAR ChIP-exo peaks and Motif 1 with respect to annotated protein coding genes. Peak were called with MACS2.

To determine whether SETMAR binds Hsmar1 ITRs *in vivo* we performed ChIP-exo on the SMF cell line. Amongst the 875 ChIP peaks, the top three over-represented sequence-motifs identified by the MEME-ChIP software were clearly related to the canonical 28 bp Hsmar1 ITR (Figure 2A and Supplementary Table S1). The most highly over-represented motif corresponds with the consensus Hsmar1 ITR, which is bound by SETMAR *in vitro* (16). Binding was validated by ChIP-qPCR of intronic ITRs in four genes in the SMF and SMFN cell lines (Supplementary Figure S1B, C). The second most over-represented motif corresponds to a divergent ITR present in a subset of Made1 elements (Figure 2A). The third motif is most similar to the core transposase binding site between bp +6 to +20 of the ITR. It also contains a similarity to the CENP-B binding site (Figure 2A). It is worth noting that CENP-B was the product of a much more ancient exaptation event involving a transposon in the same superfamily as Hsmar1 (45,46). None of the other over-represented sequence motifs identified by MEME-ChIP were related to the Hsmar1 ITR sequence or contained a conserved transcription factor binding site.

Overall, nearly half of the ChIP-exo peaks overlap or are located close to an ITR or one of the two other motifs (Figure 2B). Some 34% of the peaks are within 500 bp of an ITR, with 90% of these at <150 bp from the ITR (Supplementary Figure S1D). In a meta-profile, the maximum signal is at the center of the ITRs, which indicates direct recognition of this sequence by SETMAR (Figure 2C). Since sequence-specific DNA-binding proteins must compete with nucleosomes, we integrated the ENCODE MNase-seq metaprofiles surrounding ITRs to determine whether SETMAR could easily access its binding sites. We present data from our U2OS cell line, together with a lymphoblastoid and two human embryonic stem cell lines (Figure 2D). The profiles reveal nucleosome depletion at the center of the ITR. This is either the result of endogenous SETMAR binding or a property of the sequence, which would facilitate SETMAR binding. A phased array of nucleosome positions spreads out on either side for ∼1 kb.

We also mapped the distribution of ChIP-exo peaks with respect to the position of annotated genes (Figure 2E and F, Supplementary Figure S1E, F and G). Overall, the ChIP-exo peaks were over-represented in genic regions, presumably reflecting the accessibility of genes in chromatin. Motif 1 (ITR) was the most highly over-represented, with more than half located within 3 kb of a gene (Figure 2F).

### SETMAR is mostly associated with positive regulation of transcription

**Figure 3.**
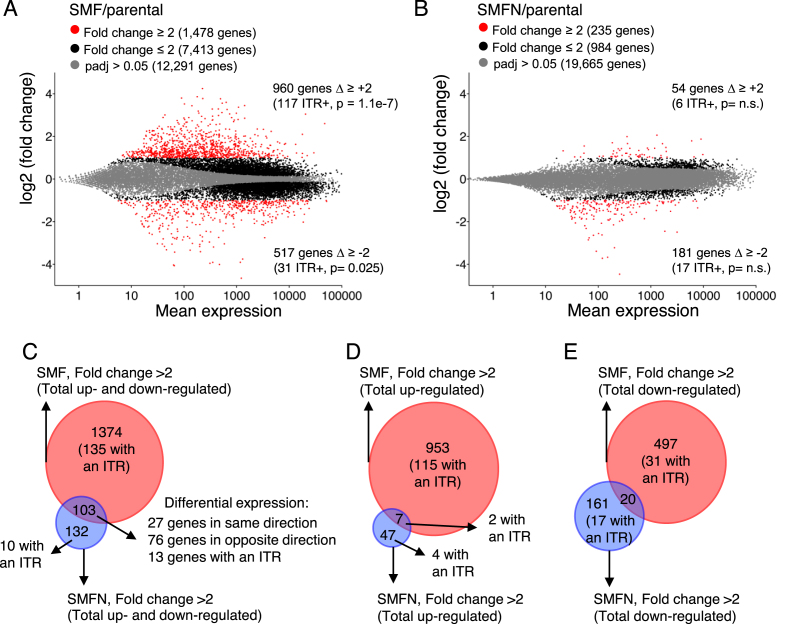
SETMAR regulates gene expression and is dependent on its methyltransferase activity. (**A** and **B**) Smear plots of RNA-seq data showing average gene expression versus log2-fold change in gene expression. Fold-change and p values are color coded as indicated. The number of genes up-regulated or down-regulated are indicated at the top and bottom of each graph, respectively. The number of genes with an ITR differentially expressed is indicated between brackets. *P*-value were determined using a hypergeometric distribution. SETMAR expression is 1372 making it the 9098th most express gene out of 16 776 genes. (**C–E**) Venn diagrams for the genes significantly differentially expressed more than ±2-fold from parts A and B.

To investigate whether SETMAR regulates the expression of genes containing an ITR located between the transcription start site (TSS) and poly(A) site, and whether the methytransferase activity is required for this function, we performed RNA-seq on the SMF and SMFN cell lines, and qRT-PCR to validate some candidates (Figure 3A, B; Supplementary Figure S2A and B; Supplementary Table S2). In SMF cells, 960 genes were up-regulated more than 2-fold compared to the parental cell line (Figure 3A). Within this group, genes with an ITR were significantly enriched (117 genes, *P* = 1.1e-7, hypergeometric distribution). In addition, some 517 genes were down-regulated more than 2-fold, of which 31 contained an ITR (*P* = 0.025, hypergeometric distribution). In the SMFN cell line, the direction of the change was reversed and the number of genes down-regulated was three times the number up-regulated (Figure 3B). This suggests that the SETMAR methylase-mutant is a dominant negative regulator. We also asked whether ITR-less genes located within 10 kb of an ITR were differentially regulated by SETMAR expression. However, this was not the case, which suggests that SETMAR does not act at a distance (465 ITR-less genes, 11 and 17 up- and down-regulated, respectively, in SMF).

Many of the differentially-regulated genes in the SMF and SMFN cell lines do not contain ITRs and the changes are likely due to secondary effects cascading through the transcriptome. To assess the extent and direction of these effects, we examined the overlap between the sets of up- and down-regulated genes (Figure 3C–E). Of the 103 genes differentially expressed >±2-fold in SMF and SMFN, 76 were changed in the opposite direction in the respective cell lines. Furthermore, of the 960 genes up-regulated in the SMF cells, only 7 responded in the same direction in SMFN, which supports a requirement for SETMAR’s methylase activity for the up-regulation of genes (Figure 3D).

### SETMAR mode of action

**Figure 4.**
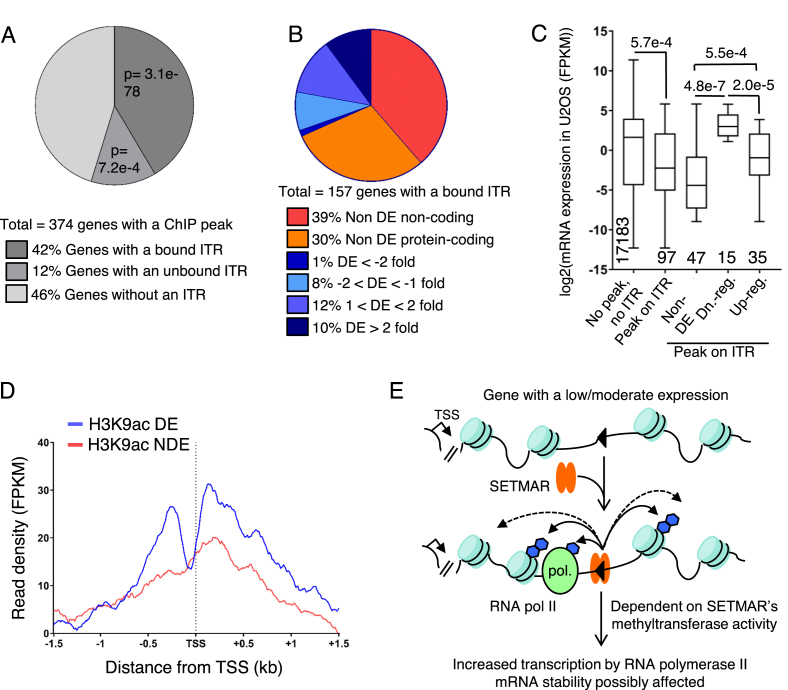
Distribution of SETMAR ChiP peaks and mode of action. (**A**) The distribution of intragenic ChIP-exo peaks with respect to the presence of intronic ITRs. 42% of genes have a ChIP-exo peak within 500 bp of an ITR. 12% of genes have a ChIP-exo peak >500 bp distant. 46% of genes with a ChIP-exo peak have no intronic copies of the ITR. *P*-values were determined using a hypergeometric distribution. (**B**) The distribution of intronic SETMAR-bound copies of the ITR with respect to non-coding and coding genes and the degree of differential expression in the SMF and SMFN cell lines. A third of the genes have their ITR bound in the ChIP-exo experiments are significantly differentially expressed in the SMF cell line. A third of the non-differentially expressed genes are non-coding and therefore have not been detected by the RNA-seq protocol used. DE, differentially expressed. (**C**) Boxplot showing the expression level of the 97 protein coding genes with a bound ITR (ChIP-exo peak <500 bp distant) compared to the vast majority of genes that have no ITR and no ChIP-exo peak. The rightmost three plots are subclasses of the 97 genes with a bound ITR. *P*-values were determined using a Mann-Whitney U test. DE, differentially expressed; Dn., down; reg., regulated. (**D**) Metaprofiles for the presence of H3K9ac marked-nucleosomes were generated for the 50 differentially expressed (DE) and 47 non-DE genes from part C using the ENCODE data for U2OS cells. TSS, transcriptional start site. (**E**) We present a model for transcriptional regulation by SETMAR. A gene is undergoing a moderate amount of transcription. An intronic ITR has positioned nucleosomes on either side owing either to SETMAR binding or the intrinsic positioning effect of the ITR sequence itself. SETMAR methylates nearby nucleosomes and/or RNA polymerase II associated factors such as snRNP70. Up-regulation of gene expression by SETMAR is dependent on its methyltransferase activity and is mediated by an increase in RNA polymerase II transcription and by a possible change in mRNA stability. Blue hexagons, methyl groups.

To gain more insight into the mechanism of SETMAR, we investigated whether SETMAR’s ITR-binding activity, which seems to not be strongly affected by nucleosomes (Figure 2D), is biased towards active or inactive genes. We first considered the set of 350 intragenic ChIP peaks, defined in Figure 2E, which are located in 374 genes (Figure 4A). There are more genes than ChIP peaks because of the presence of ChIP peaks in overlapping genes. We compare the distribution of the expression level of the genes with or without a ChIP peak in the U2OS cell line (Supplementary Figure S2C). The main difference is that 42% of the genes with a ChIP peak are amongst the set of most lowly expressed genes with FPKM values between zero and one. In contrast, only 28% of the majority of genes without a ChIP peak are expressed in this range. This may indicate that SETMAR has a preference for binding in genes with a low level of expression, or that SETMAR is displaced by transcription.

Genes with an ITR bound by SETMAR (peak <500 bp distant) were strongly over-represented amongst the genes with a ChIP peak (97 protein-coding and 61 non-coding genes, *P* = 3.1e–78, hypergeometric distribution). There were also 35 protein-coding and 10 non-coding genes with a ChIP peak >500 bp from an ITR (*P* = 7.2e–4, hypergeometric distribution) but it remains unclear whether SETMAR binding to these genes is linked to the presence of an ITR.

We next considered how the genes with an ITR bound by SETMAR responded in the SMF cell line (Figure 4B). Out of the 97 protein-coding genes bound by SETMAR, 50 genes (52%) were differentially expressed, most of which were up-regulated. Indeed, almost all of the genes that changed more than 2-fold were up-regulated. In comparison, only 44% of the 45 genes with a ChIP peak more than 500 bp from an ITR, and only 36% of the 172 genes with a ChIP peak but no ITR, were differentially expressed in the SMF cell line (Supplementary Figure S2D and E). To understand why some of the genes with a bound ITR were not differentially expressed in the SMF cell line, we compared their expression level to the average of all the others in the parental cell line (Figure 4C). The 47 non-differentially expressed genes with a bound ITR had the lowest average basal expression. This suggests that a gene must be expressed in the first instance to be further up-regulated by SETMAR. Furthermore, the up-regulated genes are on average expressed at a lower level than the down-regulated genes, and at a higher level than the non-differentially expressed genes.

To confirm that SETMAR is targeting the less-expressed genes and not the genes expressing less stable mRNAs, we re-analyzed the ENCODE ChIP-seq data for the parental U2OS cell line for the presence of H3K9ac, which is associated with active transcription (Figure 4D). The signal for the 50 differentially regulated genes with a bound ITR had a clear inflection close to the transcriptional start site. The H3K9ac signal for 47 non-differentially expressed genes was lower and noisier.

From the RNA-seq data, we know that the methyltransferase activity is required for SETMAR’s function in gene regulation. We therefore used ChIP-qPCR to determine whether SETMAR increased H3K36me2 in the vicinity of ITRs (Supplementary Figure S3A). We selected three genes with intronic ITRs that are up-regulated in the SMF cell line. In SMF cells, two of the genes had a small increase in methylation but in the third gene it decreased. We also tested two up-regulated genes lacking an ITR but only one had a significant increase in H3K36me2. Thus, even though SETMAR methyltransferase activity is required for differential expression, H3K36me2 is perhaps not the main driver.

Up-regulation in RNA-seq data might be explained by an increase in the rate of transcription and/or a higher mRNA stability. To further investigate the mechanism of SETMAR, we performed RNA polymerase II (pol II) ChIP-qPCR on LRRC55 and PBX1, which are genes with an ITR that are up-regulated in SMF (Supplementary Figure S3B). We detected an increase in the pol II level in the gene body for LRRC55 and at the transcription start site (TSS) for PBX1 in the SMF cell line compared to U2OS. We confirmed the pol II data for PBX1 by performing ChIP-qPCR against H3K4me3, an active mark specific to the TSS, and H3K36me3, an active mark in the gene body, which is added by SETD2 in human cells and requires H3K36me2 as a substrate (Supplementary Figure S3C and D). In the SMF cell line, there was a significant increase in H3K4me3 at the TSS and H3K36me3 at the first ITR. Interestingly, in the SMFN cell line there is a loss of H3K36me3 at the TSS and both ITRs, which could be due to a lower level of its precursor H3K36me2, the substrate for SETD2 (Supplementary Figure S3C and D).

We also examined the distribution of ITR with respect to the TSS and the poly(A) site and to the extent and direction of differential regulation but detected no correlation (Supplementary Figure S3E). Neither is there a significant correlation between the number of ITRs in a gene and the extent and direction of differential regulation (Supplementary Figure S3F).

## DISCUSSION

In this study we tested the hypothesis that the DNA-binding domain and methylase domain of SETMAR are both important for its biological activity. We found that modest overexpression of SETMAR causes significant changes in the transcriptome and that the set of differentially up-regulated genes is enriched for intronic copies of the cognate ITRs (*P* = 1.1e–7, hypergeometric distribution). The targeting of SETMAR to ITR sequences was confirmed by ChIP-exo and ChIP-qPCR.

RNA-seq revealed that SETMAR expression up-regulates about twice as many genes as it down-regulates (Figure 3A). In contrast, SMFN expression down-regulated about three times as many genes as it up-regulates (Figure 3B). There was little overlap between the respective sets of up- and down-regulated genes (Figure 3D, E), suggesting that SETMAR acts predominantly as a positive regulator. In the SMF cell line, we found that genes containing an ITR were over-represented amongst the differentially expressed genes, generally in the up-regulated group. ITR-less genes which are nevertheless located within 10 kb of an ITR, were under-represented amongst the set of differentially expressed genes. This indicates that for SETMAR to affect the expression of a gene, it must contain an ITR.

Within this set of ITR-containing differentially-regulated genes, there was no bias in the location of the ITR with respect to the TSS or the poly(A) site (Supplementary Figure S3E). Neither was there a correlation between the number of ITRs and the magnitude of the differential regulation between the U2OS and SMF cell lines (Supplementary Figure S3F). However, even though genes with an ITR are over-represented in the set of differentially expressed genes, most of these genes do not contain an ITR. The majority of the changes are therefore presumably due to secondary effects. If the majority of expression level changes are indirect, it raises the question of why the changes are overwhelmingly positive? Perhaps this is explained by the fact that most genes in eukaryotes are positively regulated by transcription factors. Thus, if SETMAR up-regulates a transcription factor, such as PBX1, it is more likely to initiate a cascade of up-regulation rather than down-regulation.

In Figure 4E, we present a tentative model for SETMAR’s role in genetic regulation. As an example, we illustrate an intronic copy of the Hsmar1 ITR in a gene undergoing a modest amount of transcription. SETMAR binding is facilitated by the nucleosome free region around the ITRs and Made1 elements (Figure 2D). Dimethylation of H3K36 by SETMAR may open the chromatin further. It may also recruit H3K36me2 readers, which would increase gene expression still further (not shown). Another non-exclusive possibility is the methylation of RNA polymerase II associated factors, such as snRNP70 (26), could regulate co-transcriptional processes such as mRNA splicing and thus facilitate the production of mature mRNA by decreasing premature termination of RNA polymerase II transcription. However, in the RNA-seq data there was no change in the usage of exons located within 250 bp of an ITR (data not shown). SETMAR may also methylate other chromatin readers and/or writers to promote open chromatin around the ITR (not shown). SETMAR’s mode of action remains unclear but the up-regulation of gene expression appears to be mediated directly by a higher level of transcribing RNA polymerase II, as observed for LRRC55 and PBX1 (Supplementary Figure S3B–D).

SETMAR was reported to dimethylate H3K36 *in vivo* in the vicinity of DNA double strand breaks (47). However, a different group failed to detect histone methylation by SETMAR *in vitro* (17,26). We failed to detect a significant increase in H3K36me2 in the SMF cells, either in bulk or close to bound ITRs (Figure 1F, Supplementary Figure S2C). Perhaps the high abundance of H3K36me2 in the human genome obscures the contribution of SETMAR. However, we did detect a clear and consistent reduction of H3K36me2 in the SMFN cell line (Figure 1F). In this context it is worth considering that the methylase activity may be regulated by the effects of co-factors, ITR binding or via an auto-inhibitory interaction between the SET and the post-SET domain, which might block the substrate binding pocket (48–51).

The presence of the CENP-B binding site in the Motif 3 could indicate a targeting of SETMAR to centromeric regions (Figure 2A). It has been shown that centromeric regions contain both H3K4me2 and H3K36me2 marks (52,53). However, the methyltransferases responsible are still unknown. It is therefore tempting to hypothesize that SETMAR could mediate the addition of H3K36me2 in light of its ability to bind a motif containing a CENP-B binding site.

The methylase domain of SETMAR is deeply conserved as a free-standing protein in the mammalian and avian lineages. It is pleotropic in mice and the mutants have several abnormalities in their neurology, behavior, morphology and metabolism (54). It is therefore interesting that the transposase domain was exapted at a time when many of the key genetic and adaptive changes were taking place in the anthropoid primate lineage. For example, there are 19 genes listed under the GO term Vocalization Behavior for humans. Of these, five have an ITR, with three of them bound by SETMAR, and nine are differentially expressed more than 1.5-fold in the SMF cells (Supplementary Table S2).

In humans, SETMAR is expressed at different levels in most primary tissues and cultured cells, and has been reported to play a role in DNA recombination, repair and chromosome decatenation (47,55,56). Here, we demonstrate that SETMAR misregulation is likely to have widespread effects on the transcriptome. However, the set of genes differentially-expressed >2-fold in the SMF and SMFN cells does not include well known genome-stability players, such as Ku, ligase IV, topoisomerases or the BRCA genes. Rather, the differentially expressed genes are most enriched for the KEGG ‘pathways in cancer’ and several GO terms related to organ development, cell adhesion and response to stimuli (Supplementary Table S3). This is consistent with reports that SETMAR stimulates cellular proliferation and suppresses apoptosis, and that it is up-regulated in several diseases including glioblastomas, leukemias, breast and colon cancers (19,57–59). Misregulation of SETMAR expression could therefore be involved in tumorigenesis by altering gene expression, affecting how cancer cells respond to drug treatment and promoting DNA repair through the NHEJ.

In the present study, we focused on the exogenous expression of the Flag-tagged protein because this was needed for ChIP-exo experiment to demonstrate a role for the DNA binding domain. In the future, it will be interesting to discover how the transcriptome responds to SETMAR knockdown. In this respect, it should be noted that siRNA SETMAR-silencing in a colon cancer cell line reduced expression of the SOX2 transcription factor by 60% (56). This is consistent with our RNAseq analysis in which SOX2 was down-regulated 68% in the SMFN cell line and up-regulated 2.7-fold in the SMF cells (Supplementary Table S2). This is consistent with our view of SETMAR’s general role as a positive regulator: out of the 953 genes up-regulated ≥2-fold in the SMF cells, only 7 responded in the same direction in the SMFN cells (Figure 3D).

## DATA AVAILABILITY

Sequencing data have been deposited in GEO under accession number GSE108773. The data can also be visualized here on the UCSC genome browser.

## Supplementary Material

Supplementary Data
