# Supplementary material for: Human SETMAR is a DNA sequence-specific histone-methylase with a broad effect on the transcriptome
Source: Nucleic Acids Res. 2018 Oct 17;47(1):122–33. doi: 10.1093/nar/gky937 (PMC6326780; doi:10.1093/nar/gky937)
Supplement: Supplementary Data [file gky937_supplemental_files.zip › V29 SETMAR Supplementary Figures Final.pdf]

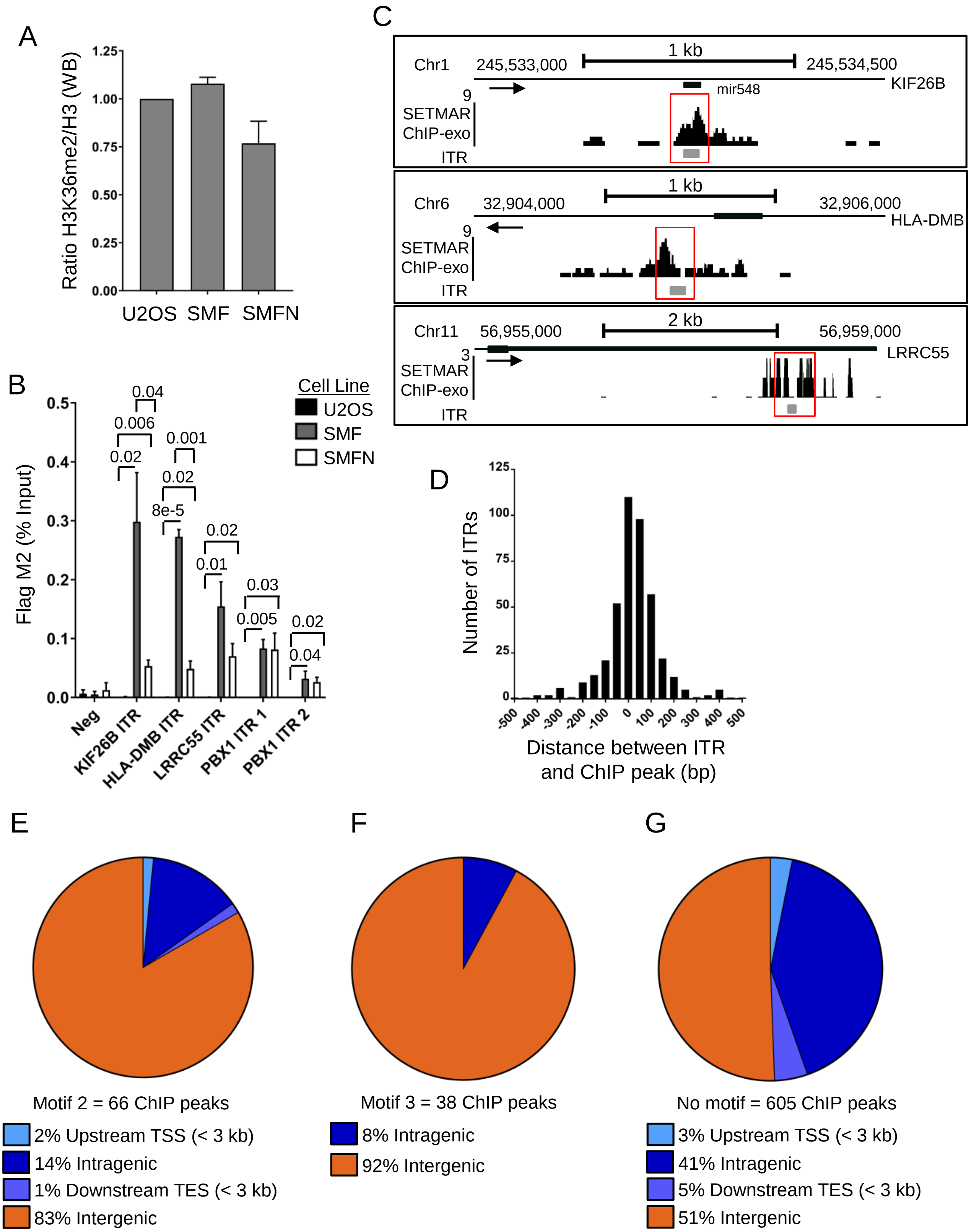

Supplementary Figure 1

Supplementary Figure 1. SETMAR binds Hsmar1 ITR in vivo

A, Quantification of the H3 and H3K36me2 level as determined by western blotting in the U2OS, SMF and SMFN cell line. The H3K36me2/H3 ratio of three biological replicates is shown.
B, Four genes identified by the ChIP-exo as having SETMAR-bound intronic-copies of the ITR were validated by ChIP-qPCR in the indicated cell lines. After crosslinking, FLAG-tagged SETMAR was pull down using the M2 antibody. Neg. corresponds to a genomic region without an ITR. The ChIPs were in triplicate and error bars represent $\pm$ S.E.M. P-value were determined using an unpaired t-test.

C, Representative genome browser tracks for the genes in part A indicating the read coverage from the ChIP-exo experiment in Figure 2. The position of the Hsmar1 ITR or Made1 element is indicated within the red box.

D, Distribution of the distance of the 297 ChIP peak overlapping or located close to an ITR. The distance was calculated as the number of bp between the centre of the ITR and the centre of the ChIP peak. 90% of the 297 ChIP peaks are located at less than 150 bp of the centre of an ITR.
E, F & G, Genomic distribution of Motifs 2 & 3 from Figure 2A, and the distribution of ChIP-exo peaks without an enriched motif.

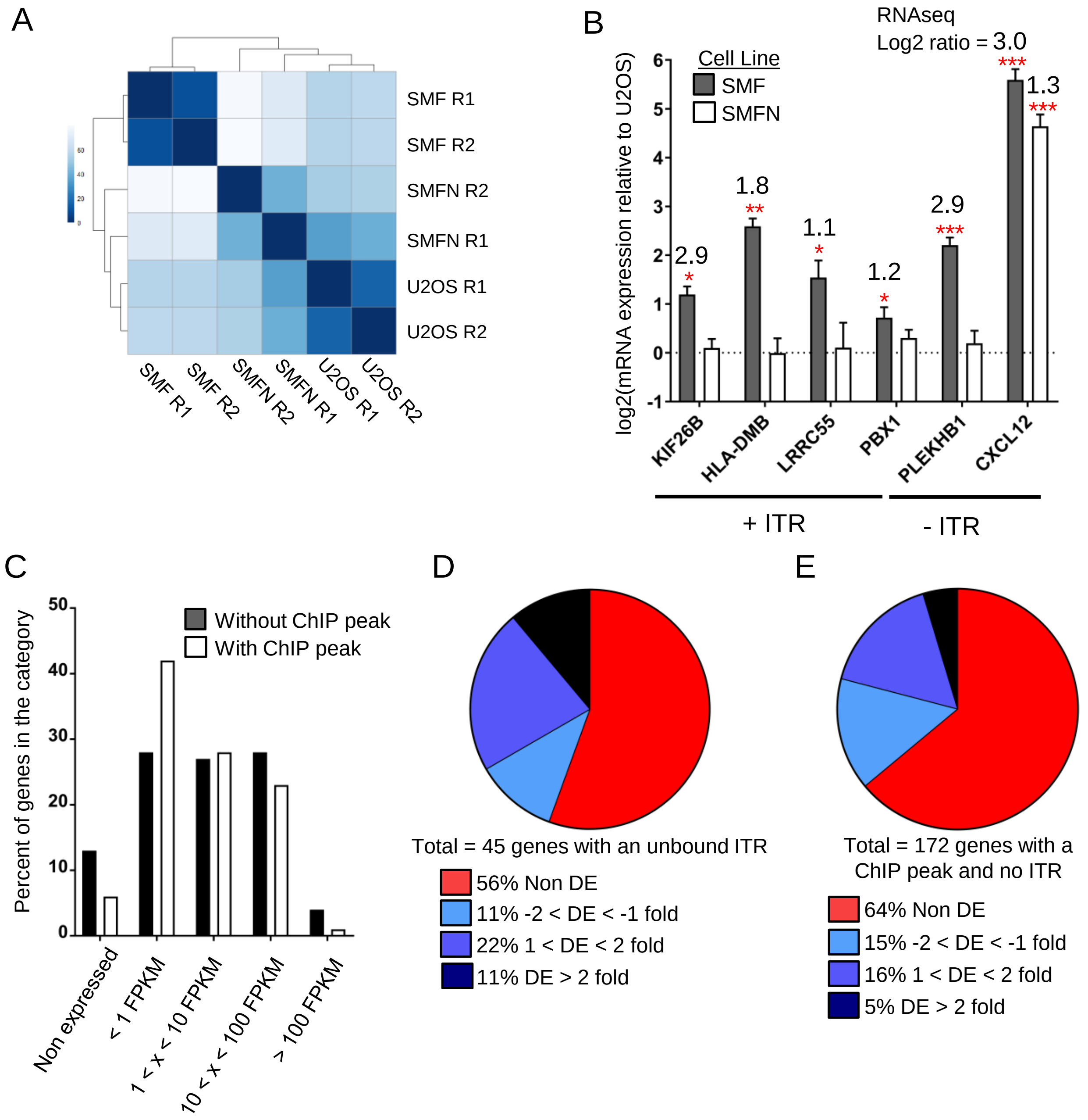

Supplementary Figure 2

Supplementary Figure 2. Validation of RNA-seq

A, Hierarchical clustering analyses of the duplicate DEseq2 rlog-normalized RNA-seq data in the indicated cell lines. Color code (from white to dark blue) refers to the distance metric used for clustering (dark blue corresponds to the maximum of correlation values).

B, The differential regulation of six genes in the indicated cell lines was validated by qRT-PCR. The log2-fold change in the RNA-seq data is indicated above each bar graph. P-values were determined using an unpaired t-test, * $p < 0.05$, ** $p < 0.01$, *** $p < 0.001$. The qRT-PCR were in triplicate and error bars represent ± S.E.M.

C, Distribution of the expression level in the U2OS cell line of the 247 genes with a ChIP peak or of the 18,990 genes without a ChIP peak.

D, E, Degree of differential expression in the SMF and SMFN cell lines for the set of genes with a ChIP peak more than 500 bp away from an ITR (D) or for the set of genes with a ChIP peak and no ITR (E).

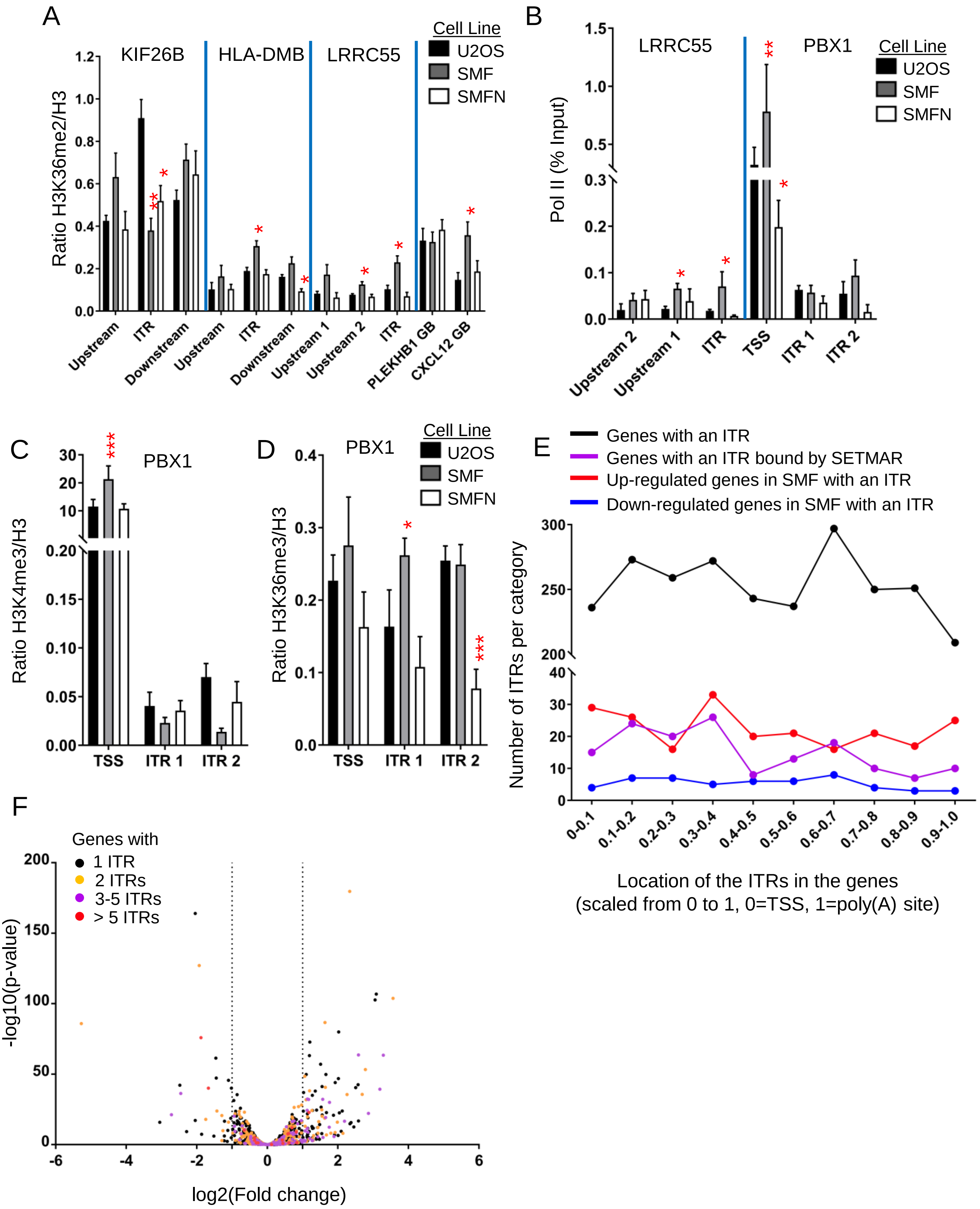

Supplementary Figure 3

Supplementary Figure 3. Transcriptional and post-transcriptional effect of SETMAR expression

A, ChIP-qPCR was performed using antibodies for H3 and H3K36me2 in the five genes from part B. Regions of the gene body (GB) some distance upstream and downstream of the ITR were used as a control. PLEKHB1 and CXCL12 represent a second control since these genes do not have an ITR but are up-regulated in SMF. CXCL12 is also up-regulated in the SMN cell line. P-value were determined using an unpaired t-test, * $p < 0.05$, ** $p < 0.01$. Experiments were in triplicate and error bars represent ± S.E.M.

B, ChIP-qPCR was performed using antibodies for RNA polymerase II in two upregulated genes in SMF containing an ITR. P-value were determined using an unpaired t-test, * $p < 0.05$, ** $p < 0.01$. Experiments were in triplicate and error bars represent ± S.E.M.

C, ChIP-qPCR was performed using antibodies for H3 and H3K4me3 in the candidate gene PBX1 from part D. P-value were determined using an unpaired t-test, *** $p < 0.001$. Experiments were in triplicate and error bars represent ± S.E.M.

D, ChIP-qPCR was performed using antibodies for H3 and H3K36me3 in the candidate gene PBX1 from part D. P-value were determined using an unpaired t-test, * $p < 0.05$, *** $p < 0.001$. Experiments were in triplicate and error bars represent ± S.E.M.

E, Distribution of the ITRs across the scaled gene length for all the genes with an ITR, the genes with an ITR bound by SETMAR or the up- and down-regulated genes in the SMF cell line. Each gene has been divided in 10 segments with 0 and 1 representing the TSS and the poly(A) site, respectively.

F, Volcano plot of differentially expressed genes with an ITR in the SMF cell line. No difference in the fold change is observed between genes with a single, two or more ITRs.
